# Lower limb rehabilitation using modified constraint-induced movement therapy and motor relearning program on balance and gait in sub-acute hemiplegic stroke: a comparative study

**DOI:** 10.12688/f1000research.138127.1

**Published:** 2023-09-01

**Authors:** Nitika S. Chavan, Raghumahanti Raghuveer

**Affiliations:** 1 Department of Neurophysiotherapy, Ravi Nair Physiotherapy College, Datta Meghe Institute of Higher Education and Research (Deemed to be University), Wardha, Maharashtra, 442004, India

**Keywords:** Hemiplegic Stroke, Modified constraint-induced movement therapy, Motor relearning program, Berg balance scale, Dynamic gait index

## Abstract

**Background:** A stroke is described by the World Health Organization as “a clinical syndrome with rapidly developing symptoms that consist of a focal (or global, in a situation of coma) disruption of cerebral function that lasts more than 24 hours or leads to mortality without a known cause other than a vascular origin”. Stroke is the most prevalent cause of impairment and mortality on a global scale. Modified constraint-induced movement therapy (mCIMT) is an approach to therapy for motor disabilities that involves constraining the movements of the nonparetic limb, diligent practice and behaviour modification to extend the time the paretic limb is utilized for daily tasks. The motor relearning program (MRP) method involves many aspects of motor learning theory and is helpful in providing instructions for retraining practical skills (including walking, standing and sitting in balance and transferring abilities). So, the objective of this study is to assess the impact of the MRP and mCIMT on balance and gait in sub-acute hemiplegic stroke patients.

**Methods:** In this study, each group will consist of 17 people in total. The randomization procedure will be conducted using a computer-generated random number system. For sample distribution, we will use the sequentially numbered opaque sealed envelope technique. Outcome measures will be as follows: Berg balance scale, Dynamic gait index, Trunk impairment scale, Functional reach test, 10 Meter walk test and Fall efficacy test. Each patient will be evaluated prior to and during treatment at baseline and six weeks later.

**Conclusions:** There is sufficient evidence to derive the conclusion that the functional mobility and balance of stroke victims can be improved with physiotherapy. Therefore, this study will try to seek the comparison of mCIMT and MRP in sub-acute stroke subjects and compare the two regimes to determine which one will be superior.

**Registration:** CTRI ( CTRI/2023/05/052674; 16/05/2023).

## Introduction

Stroke, which is deemed by the World Health Organization (WHO) to be the “quickly growing clinical evidence of a permanent (or worldwide) brain function impairment with sign and symptoms lasting 24 hours or more or causing mortality with no apparent cause other than the vascular origin”. ^1^ Stroke incidence ranges from 105 to 152/100,000 per year, making it the third most prevalent cause of impairment and the second-leading cause of mortality worldwide. ^2^^,^^3^ In India, the prevalence of stroke considerably rises with age; compared to younger persons, the risk is higher for those over 55 years old and lower for those between 45 and 54 years old (0.80% *vs.* 1.97%, respectively). Stroke risk is higher in men than in women. African Americans, Hispanics, and Americans have a higher risk of stroke compared to other races. Americans are more likely to have a stroke. ^4^ Stroke is divided into two distinct subtypes based on its aetiology: ischemic and haemorrhagic. The ischemic type of stroke, which affects 85% of patients, arises whenever the vascularity of the brain tissue is damaged as the blood clot blocks or plugs a blood vessel in the brain. However, 15% of acute strokes are haemorrhagic strokes, which are brought on by a burst blood vessel or an abrupt haemorrhage. Subarachnoid and intracerebral haemorrhage are the two main forms of haemorrhagic attacks. ^5^

There are numerous causes of stroke. Common risk factors include smoking (12%), heredity, hypertension, diabetes mellitus, high cholesterolemia (15%) and lack of physical activity. ^4^ Alcohol consumption that is mild to moderate carries a slightly lower risk of ischemic stroke, however higher drinking significantly boosts the risk. ^6^ Common stroke problems include gait impairment that makes it difficult to maintain postural alignment as well as stiffness, fatigue, and loss of balance on the affected side. One of the most frequent problems following a stroke is paralysis. Usually, opposing the brain side of the body that has been harmed by a stroke undergoes the paralysis. It can affect the overall side of the body, the face, upper limb, lower limb or even one of the legs. Hemiplegia refers to this type of unilateral paralysis. There are several anatomical and functional abnormalities that are followed by hemiplegia. These people frequently have functional mobility and balance issues as a result of their movement paralysis. ^7^ Without using more resources, a planned program can considerably raise documented patient activity levels during inpatient stroke therapy.

The main goal of stroke treatment is to promote activity in daily life (ADL) independence while restoring function. ^8^ There are numerous physiotherapy approach treatments for stroke, including body weight supported treadmill training, electromechanical-assisted training, virtual reality therapy, constraint-induced movement therapy, motor relearning program, robot-assisted training, mirror therapy and proprioceptive neuromuscular facilitation. All methods for restoring voluntary control and mobility after a stroke operates according to its own principles. ^9^ Major studies have revealed that muscle weakness is the main thing preventing physical function from recovering. ^9^ Conventional exercises are ones that are performed all around the world. These include exercises such as passive motion, exercise at the gym, gait training, electrical stimulation, and functional re-education. ^10^ Modified constraint-induced movement therapy (mCIMT) is a type of therapeutic intervention for those with motor disabilities. The established method for treating the paretic upper limb enhances the functional usage by preventing “learned disuse” that occurs after a stroke. ^11^^,^^12^

Three main elements of mCIMT include the use of repetitive, structured, practice-intensive therapy in the more paretic limb, constraint of the non-paretic limb and a variety of behavioural strategies that translate improvements made in a clinical setting to everyday life. Recently, mCIMT has also been used to the paretic lower limb with the goal of improving neurological function and prevent “learned misuse”. One of the rehabilitation techniques utilised predominantly with the post-stroke population is the motor relearning program (MRP). This technique incorporates several elements of motor learning theory and includes practical advice for retraining practical abilities (such as a balanced sitting position, sitting and standing, skill transfer, and walking). ^13^ With good feedback and practise, this approach focuses on the growth of active movement control and task-specific learning. It is less frequently encountered to use facilitation tactics in preference for verbal direction, demonstrations and manual recommendation. ^14^

Three main elements of mCIMT include the use of repetitive, structured, practice-intensive therapy in the more paretic limb, constraint of the non-paretic limb and a variety of behavioural strategies that translate improvements made in a clinical setting to everyday life. Recently, mCIMT has also been used to the paretic lower limb with the goal of improving neurological function and prevent “learned misuse”. One of the rehabilitation techniques utilised predominantly with the post-stroke population is the motor relearning program (MRP). This technique incorporates several elements of motor learning theory and includes practical advice for retraining practical abilities (such as a balanced sitting position, sitting and standing, skill transfer, and walking). ^13^ With good feedback and practise, this approach focuses on the growth of active movement control and task-specific learning. It is less frequently encountered to use facilitation tactics in preference for verbal direction, demonstrations and manual recommendation. ^14^

### Aims and objectives

1. To study the effect of mCIMT in addition to conventional therapy on balance (using the Berg Balance Scale (BBS)) and gait (using the Dynamic Gait Index (DGI)) in sub-acute hemiplegic stroke.
2. To study the effectiveness of MRP along with conventional therapy on balance (BBS) and gait (DGI) in sub-acute hemiplegic stroke.
3. To compare mCIMT and the MRP in improving balance and gait in sub-acute hemiplegic stroke patients along with conventional therapy.

### Trial design

A single-centre, two arm, parallel group, comparative open labelled superiority trial will be conducted.

## Methods

### Protocol

The model consent form, data collection form and schedule of enrolment, interventions and assessments can be found as *Extended data.* ^24^ This protocol adheres to the SPIRIT guidelines. ^25^

**Figure 1.**
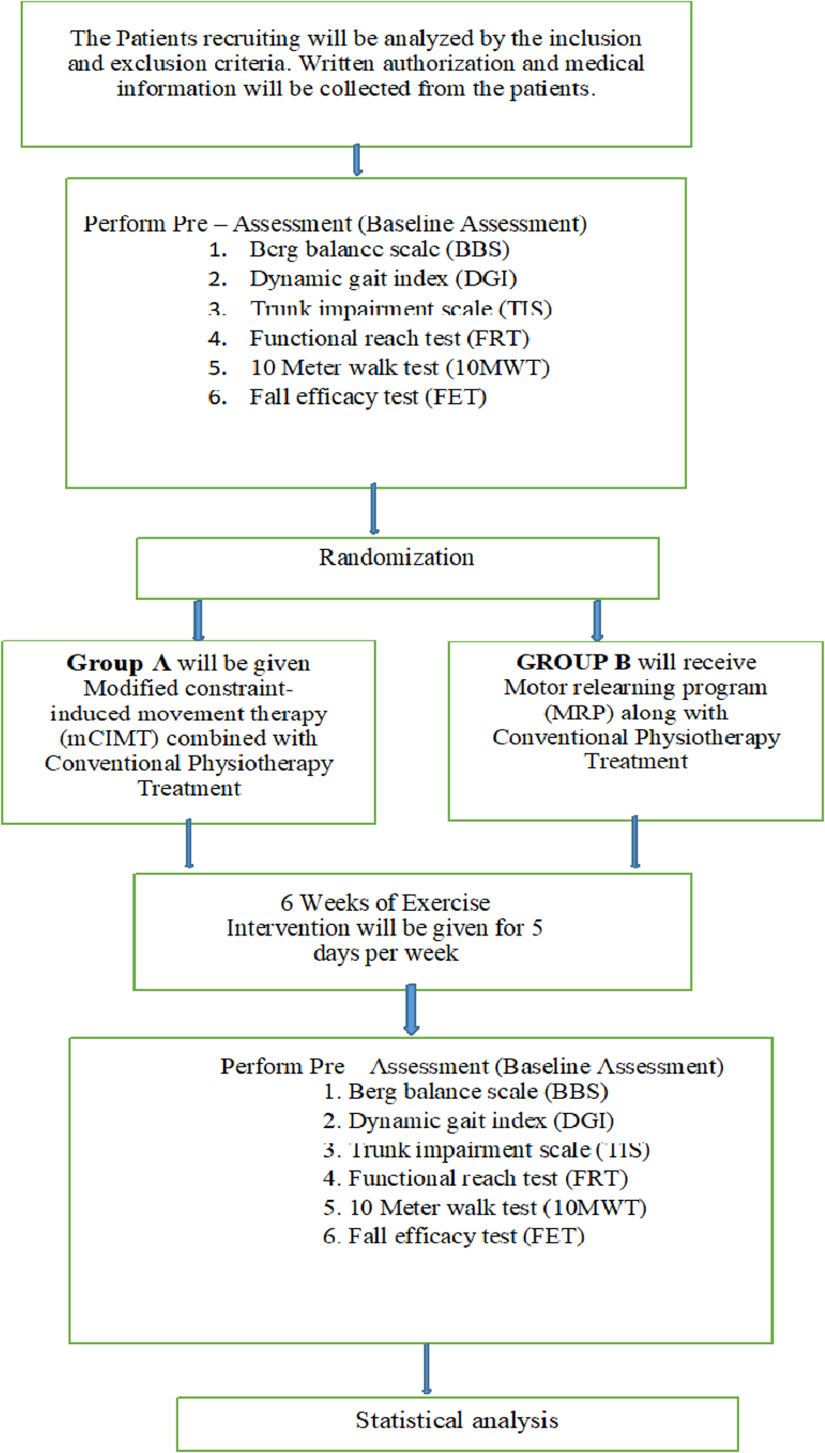
Summary of study process.

**Figure 2.**
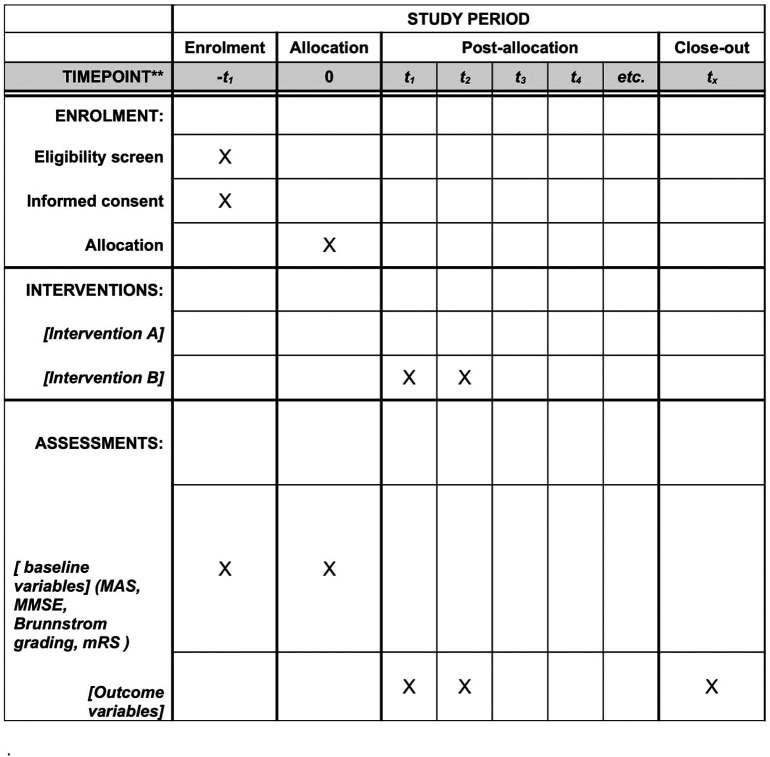
Schedule of enrolment, interventions and assessments.

After gaining clearance from the Institutional Ethics Committee (IEC) of Datta Meghe Institute of Higher Education and Research (Deemed to be University), participants will be enrolled from the Physiotherapy Outpatient Department of Acharya Vinoba Bhave Rural Hospital Sawangi, Meghe, Wardha, Maharashtra. A total of 34 participants will be made aware of the study’s objectives and procedures and will be asked to sign written patient consent forms. All hemiplegic stroke patients who meet the inclusion and exclusion parameter will be included in the study. They will be further separated into Group A and Group B by means of simple random selection. Each group will consist of 17 people in total. The randomization procedure will be conducted using a computer-generated random number system. For sample distribution, we will use the sequentially numbered opaque sealed envelope technique. There will be no blinding procedure in this study. The principal investigator will generate allocation, enrol participants and assign participants to interventions. A departmental committee made up of the Post Graduate (PG), Guide, the Head of Department (HOD), the Principal of the Ravi Nair Physiotherapy College (RNPC) and a member of the Research Guidance Cell will oversee the project. Through routine treatment sessions, we will make sure that the patients follow the suggested treatment plan closely. If necessary, individuals will receive counselling or telephone reminders about their therapy appointments. Prior to and following the analysis, the outcome measures will be assessed. The study’s outcome indicators are as follows: The BBS, DGI, Trunk Impairment Scale (TIS), Functional Reach Test (FRT), 10 Meter Walk Test (10MWT) and Fall Efficacy Scale (FES) along with conventional treatment for balance and gait. mCIMT will be given in Group A in addition to conventional therapy, whereas Group B will receive the MRP in addition to conventional therapy. Figure 1 and Figure 2 depict the study design.

### Inclusion criteria

Patients will be included if they fulfil the following inclusion criteria: i) Sub-acute stroke in both men and women between the ages of 45 and 65 years; ii) Mini-Mental State Examination score ≥24; iii) Modified Rankin Scale ≤3; iv) patients with Brunnstrom stage for hand and leg ≥4; v) Modified Ashworth Scale ≤2; and vi) be able to comprehend and adhere to instructions.

### Exclusion criteria

The exclusion criteria will be as follows: i) Individuals who are experiencing balance problems as a result of neurological conditions other than stroke (for instance cerebellar impairment, inner ear dysfunction, or Parkinson’s disease); ii) individuals suffering from unstable angina, symptomatic heart failure, or uncontrolled hypertension; iii) acute stroke (flaccid stage); iv) physician determined unstable cardiovascular conditions; v) the patient has been diagnosed as having organ failure, including the heart, kidneys, and lungs; and iv) patients with any traumatic musculoskeletal injury to lower limbs.

### Interventions

*Criteria for discontinuing allocated interventions for a given trial participant*

If any patients in any of the allocated groups develop any complication ( *e.g.*, deep venous thrombosis), their participation will be discontinued in the study. The patients will then receive appropriate care and offered outpatient rehabilitation as needed. Study participants will be retained in the trial (unless they withdraw their consent) to enable follow-up data collection and to prevent missing data.

*Adherence and concomitant care*

During the first consultation, the importance of following the study guidelines and adhering to the allocated group will be highlighted. Exercise adherence will be recorded as a percentage of completed exercises during the first follow up (after the six-week intervention) for both groups. Exercise adherence will be taken into consideration when performing the pre-protocol analysis.

All enrolled patients will be encouraged to contact the principal investigator directly if experiencing problems related to their allocated group intervention. The principal investigator will then fill out a standardized form at these calls. Patients can use medicines and nutritional products as needed, prescribed by their physician. To avoid study-contamination, patients will be asked to adhere to the allocated rehabilitation intervention and not to seek alternative health-care services during the course of the study. Patients will be advised to contact their general practitioner or the principal investigator as needed.

The physiotherapy intervention will be given to Groups A and B for six weeks. The intervention will be provided for 60 minutes, five days each week. Both Group A and Group B will receive conventional treatment. Group A will receive mCIMT along with the conventional therapy, while Group B will receive the motor relearning program along with the conventional therapy. Treatment will be provided to both groups for six weeks.

*Modified constraint-induced movement therapy (mCIMT)*

Exercises will be given to participants that have a functional focus and will be under supervision. The affected lower extremity will be the focus of these exercises. Based on the desired movements, the therapist (principal investigator) recommended these exercises.

The 30-minute mCIMT approach will consist of the following movements: Transfer package (10 minutes per session), side stepping, ball kicking, stair climbing, and knee control on a step.

*Motor Relearning program (MRP)*

Participants receive task-specific guidance based on the MRP. This method will be applied for 30 minutes. The actions will be practiced in both sitting and standing and should target an individual’s particular deficits: supine to side-lying to sitting, looking up at ceiling (ensure that centre of body mass does not move back when head is tilted back), without moving one’s feet, turning to gaze over each other’s shoulders and scanning the surroundings for specific items, reaching motions in multiple directions while moving the head and trunk, scooting in bed, altering the base of support (standing with your feet together, in tandem, with one foot on a step, or on one leg), squatting to pick up an object and cross over stepping.

*Conventional therapy*

Conventional therapy includes: i) Passive and active assisted range of motion exercises for upper and lower extremities; ii) stretching exercises for flexor synergy component shown in Table 1; iii) strengthening exercises with the help of THERABAND shown in Table 2; iv) balance training including practicing reaching beyond arm’s length while sitting and standing; and v) walking training that includes challenge to dynamic balance ( *e.g.*, obstacle courses).

**Table 1.** Stretching exercises for upper extremities, pelvic position and lower extremities.

Action of the muscles	Major muscles targeted	Procedure
• Scapular retraction or hyperextension, shoulder abduction, elbow flexion, forearm supination, wrist and finger flexion • Pelvic retraction • Hip flexion, abduction, external rotation, knee flexion, ankle dorsiflexion, inversion	• Pectoralis major, Pectoralis minor, Latissimus dorsi, Biceps brachii, brachioradialis, Supinator muscle, Flexor digitorum profundas, Flexor digitorum superficialis and Palmaris longus • Piriformis and Thoracolumbar fascia • Gluteus minimus, Gluteus medius, Psoas major, Iliacus sartarius, Rectus femoris, Hamstring, Tibialis anterior	Patient position: Supine and sitting Therapist position: Standing Mode of stretching: Manual stretching, slow repetition, continuous stretch Doses: 3 repetitions with 30 seconds hold

**Table 2.** Strengthening exercises for upper extremities, pelvic position and lower extremities.

Action of the muscles	Major muscles targeted	Procedure
• Scapular retraction or hyperextension, shoulder abduction, elbow flexion, forearm supination, wrist and finger flexion • Pelvic retraction • Hip flexion, abduction, external rotation, knee flexion, ankle dorsiflexion, inversion.	• Rhomboid, Latissimus dorsi muscle, Deltoid, Triceps, Pronator teres, Extensor digitorium, Extensor digiti minimi. • Quadratus lumborum • Adductor mangus, Adductor longus, Adductor brevis, Gracillis, Gluteus maximus, Quadriceps, Gastrocnemius	Patient position: Supine and sitting Therapist position: Standing Progression: Strengthening exercises will be given with THERABAND (Yellow, Red and Green colour).

### Outcome measures

Time frames at baseline and six weeks later.

*Primary outcomes*

1. Change in Berg Balance Scale (BBS)
  The BBS is a scale with 14 items that is frequently used and is a standardized balance assessment that uses observation to quantitatively assess a patient’s capacity for balance, either statically or while engaging in a variety of planned routine daily tasks. For evaluating static and dynamic balance following a stroke, The inter-rater and intra-rater reliability of BBS for the patients with stroke is 0.97 and 0.98, respectively. ^15^ Each item on the scale will be given a score between 0 and 4, giving the patient an overall ranking of 56. A score of 0 means you didn’t complete the project, and a score of 4 means you did it independently. ^16^
2. Change in Dynamic Gait Index (DGI)
  The goal of the DGI is to examine dynamic balance while in motion. Eight of the tasks ask participants to keep their balance when walking normally and while doing so in various conditions (such as adjusting their speed, turning their heads to go around obstacles, pivoting around and climbing steps). Each item is given a score between 0 and 3 points, with a maximum score of 24. A higher DGI score indicates greater independent functional mobility. The DGI has been shown to yield ratios of subject variability to total variability with excellent interrater reliability 0.96 and test-retest reliability 0.98. ^17^

*Secondary outcomes*

1. Change in Trunk Impairment Scale (TIS)
  The TIS assesses post-stroke trunk movement, static and dynamic sitting balance, and trunk movement coordination. The TIS is used to analyse motor dysfunction of the trunk after a stroke, static and dynamic seated balance and coordination of trunk movement. Overall reliability for clinical care and research in the TIS, 0.96. ^18^
2. Change in Functional Reach Test (FRT)
  Utilising a yardstick fastened to the wall at the acromion’s height, functional reach is determined. Functional reach has been shown to exhibit test-retest reliability, interobserver consistency, criterion validity, and concurrent concept validity in earlier investigations. Additionally, it has been demonstrated that in patients undergoing rehabilitation, functional reach is sensitive to clinically significant changes in balance. ^19^
3. Change in 10 Meter Walk Test (10MWT)
  Ambulation speed is a crucial component of gait and is frequently utilised in clinical and research settings as an objective indicator of functional mobility. In healthy older adults, gait speed can also be determined using the 10MWT. Therefore, to obtain the most accurate clinical evaluation of walking speed, we advise performing the 10MWT. ^20^
4. Change in Fall Efficacy Scale (FES)
  The FES International, a tool designed to measure patient’s fears about falling, has strong psychometric qualities. The 16 items on the Persian FES were factor analysed. The Persian FES showed outstanding test-retest reliability, according to the results. In stroke patients, FES showed that the assessment of fear of fall was appropriate and acceptable. Concerns of falling during physical and social activities could be addressed using the FES. Additionally, it can be applied in a clinical context to identify individuals who have a fear of falling. ^21^

### Sample size calculation

We estimated the sample size using a power analysis with 80% power and 5% Type 1 error for the main variables. The BBS score, in comparison to the difference in mean score pre and post treatment for mCIMT group from baseline to end visits. For the study, we used the previously determined effect size difference in percentage from the RCT. ^22^ Formula using mean difference:

$$n1=n2=2\frac{{\left(Z_{\alpha }+Z_{\beta }\right)}^{2}\sigma^{2}}{{\left(\delta \right)}^{2}}$$

Primary Variable (BBS)

Mean ± SD (Pre) result on BBS for mCIMT = 48.8 ± 4.7

Mean ± SD (Post) result on BBS for mCIMT = 52.5 ± 3.5

Difference = 3.7

Pooled std. dev. = (4.7 + 3.5)/2 = 4.1

$$\text{N1}=2^{*}\left[{\left(1.64+0.84\right)}^{2}{\left(4.1\right)}^{2}\right]/{\left(3.7\right)}^{2}=15$$

Considering 10% dropout = 2

Total samples required = 15 + 2 = 17 per group.

Total sample size required = 15

Notations:

$$Z_{\alpha }=\text{1.64 bat 5\% l.o.s. at both sides total 10\% type I error}$$

α=Type I error at 5%

$Z_{\beta }=0.84\;\left(1-\beta \right)=\text{Power at}\;80\%$

σ=std.dev=0.5pooledstd.dev

### Data collection methods

Before group allocation, screening of the participants as per the inclusion and exclusion criteria will be carried out. This will be followed by baseline assessment of the outcome measures as mentioned. All participants will undergo the randomization process and are allocated into either group A or Group B. The intervention will be administered for an hour daily, five days per week for six weeks. The post intervention data for the outcomes will be recorded after the last intervention session day. The data will be compiled and analysed for studying the results. The study interventions will be administered by the principal investigator under the supervision of the guide throughout the duration of the study. The patients will be instructed and reminded of the sessions in advance through messages sent to their mobile phones so that adherence to the study is ensured. Furthermore, if the patient misses their sessions due to any reason, the standby days from the week will be utilized such that the patient receives a total of 30 sessions of intervention in the six week duration.

As per in the sample size calculation we have given a 10% dropout rate so that it does not interfere with the results of our study.

The data collection forms can be found as *Extended data.* ^24^

### Data management

The evaluation data will be derived from a pre-set spreadsheet with varied baseline attributes. The research data will be stored in a safe database. Hard copies of evaluation forms, signed informed consent forms, and other non-electronic documents will be safely preserved in the study setting. Every month until the trial is over, a complete backup of the data entries will be performed. The lead investigators will supervise the data gathering and reporting processes. The accuracy of the research papers must be properly reviewed. At the end of the study, the Excel spreadsheet will be published and delivered to the statistician for the necessary analysis. A checklist can be used to avoid data loss due to ineffective staff processes. Due to the thorough follow-up assessment of this trial, participant retention and completion of follow-up assessments are anticipated to be quite high. The patients in this trial will be invited to follow-up exams after six weeks.

### Statistical analysis

Samples taken in accordance with the protocol and recorded on a basis that includes all relevant characteristics. Dataset of outcome variables including demographic, physical, laboratory and vitals information will be collected and tabulated in Excel, and will be analysed using R-Software version 4.3. Demographic variables such as age and gender and physical examination (height, weight, BMI), laboratory parameters (haemoglobin, Erythrocyte Sedimentation Rate (ESR), Liver Function Test (LFT), Kidney Function Test (KFT)), and vitals (respiratory rate, pulse rate, systolic blood pressure, diastolic blood pressure and SpO _2_ levels) will be collected. Outcome variables such as BBS, DGI, TIS, 10MWT, FRT and FES will be presented over descriptive statistics with categorical tabulation for frequency and percentage, while quantitative measurements with minimum, maximum, mean and standard deviation.

The primary outcome variables will include the BBS for measuring balance and the DGI for measuring gait outcome variables, the secondary outcome variables include the TIS, FRT, 10MWT and FES. Variables as primary and secondary parameters for their measuring assessment at different time visits will be assessed to find the significance in mean at 5% level of significance at P-value < 0.05. Statistical analysis will be performed for hypothesis testing for paired pre and post analysis using a paired t-test for normal data or non-parametric test such as Wilcoxon signed-rank test for non-normal data. Similarly, an unpaired t-test will be used for two independent groups for the change in parameters readings of primary and secondary variables at two different visits.

Data over the outcome variables will be analysed for normality (normal distribution) using Kolmogorov–Smirnov test. If data results over non normal distribution then alternate non parametric test will be used.

Confounding variables will be analysed with generalised linear model to find the random and fixed effects over the outcome variables. Association analysis for finding significance of cofounding parameters will be examined by using Chi-squared test or Fisher’s exact test or by using multi-variant analysis.

Full analysis set for the values of outcome variables will be analysed for the results missing data will be covered with imputation method by calculating the mean and median over the existing data.

### Monitoring

*Data monitoring*

We will have a data monitoring committee lead by the PI for maintaining and integrating of the data.

*Harms*

The whole procedure is going to held under the supervision of clinicians and departmental committee During the trial, harms and adverse event will be immediately reported to the committee. The final dataset will be uploaded to the institutional research website and will be accessible to concerned authorities.

*Auditing*

Every month, auditing of the trial is going to be conducted. Any deviation from the protocol will be documented and will be addressed.

## Ethics and dissemination

### Ethical considerations

Datta Meghe Institute of Higher Education and Research, Sawangi Wardha granted ethical approval (date, 21/03/2023) (reference number, DMIHER (DU)/IEC/2023/813). This protocol has been registered on CTRI (trial registration number, CTRI/2023/05/052674; registration date, 16/05/2023).

### Protocol amendments

The study has been approved by the Scrutiny Committee at the college level on 01/03/2023 and by the IEC at the university level on 21/03/2023 following which, CTRI registration was also done. As per the suggestions through these committees the study has already been modified and approved. So further changes could not be carried out.

### Consent

Members of the trial committee will provide the consent form to the participants in the trial and will inform and explain all the potential benefits and risks to the participants.

### Confidentiality

The study program will be elaborated to the participant and one of their relatives, and the principal investigator will take personal information as a part of procedure. The consent form will include the confidentiality statement and signatures of the principal investigator, patient and two witnesses. Whenever it’s necessary to divulge details for the study, consent will be obtained from the patient with complete assurance of their confidentiality.

### Access to data

The Principal Investigator will have access to the final trial datasheet.

### Ancillary and post-trial care

The whole procedure is going to held under the supervision of clinicians and the departmental committee *i.e.*, Guide, Head of department, Principal and member of Research Guidance Cell.

After the trial session, the participations are going to be under supervision for about four weeks so that if there will be any harm, the Principal Investigator will take care of the participants.

### Dissemination policy

We will present the work in International Conference Proceedings and publish in an indexed journal.

**Study status**

The study is yet to begin.

## Discussion

The study’s primary objective is to determine how well mCIMT and MRP work to treat balance and gait issues. These cause a rise in the risk of falling, which further reduces the functional independence of those who had sub-acute stroke. In a previous study, the experimental group received mCIMT for two weeks, while the control group received neurodevelopmental therapy (NDT). The findings showed that mCIMT for lower limb had a superior impact on improving motor function compared with NDT. ^22^ Using a randomized controlled trial, the study was done to determine how well the MRP worked to improve balance and upright mobility in sub-acute stroke patients. The results suggested that MRP showed a good impact on post-stroke survivors with the experimental group receiving MRP in addition to conventional physical therapy (RPT) for a duration of five days per week for eight weeks. ^23^ Both interventions were found to be beneficial in hemiplegic stroke when compared with a negative control group. But there is dearth of literature reflecting the superiority of either of these interventions when compared with each other. Therefore, our study will compare the two regimes to evaluate which is more successful while looking at the effectiveness of these therapies in sub-acute stroke patients.

## Data Availability

No data are associated with this article. Zenodo: Extended data - Nitika.docx, https://doi.org/10.5281/zenodo.8172474. ^24^ This project contains the following extended data:
- Model consent form - Data collection form - Schedule of enrolment, interventions and assessments. Zenodo: SPIRIT- Nitika.docx, https://doi.org/10.5281/zenodo.8172339. ^25^ Data are available under the terms of the Creative Commons Attribution 4.0 International license (CC-BY 4.0).
