# Supplementary material for: Lower limb rehabilitation using modified constraint-induced movement therapy and motor relearning program on balance and gait in sub-acute hemiplegic stroke: a comparative study
Source: F1000Res. 2024 Mar 28;12:1098. Originally published 2023 Sep 1. [Version 2] doi: 10.12688/f1000research.138127.2 (PMC11406135; doi:10.12688/f1000research.138127.2)
Supplement: Peer review history

# Reviewer report: Reviewer response for version 2

**DOI:** 10.5256/f1000research.164215.r260278

**Published:** 2024-04-29

**Authors:** Ingela Marklund

**Affiliations:** 1 Umeå universitet, Umeå, Sweden; 2 Centre for Clinical Research and Education, Region Värmland, Karlstad, Sweden

I have read the revised manuscript with the tracked changes marked and none of the comments I made before have been answered or taken into account not even the paragraph that is duplicated has been removed.

Is the study design appropriate for the research question?

Yes

Is the rationale for, and objectives of, the study clearly described?

Partly

Are sufficient details of the methods provided to allow replication by others?

No

Are the datasets clearly presented in a useable and accessible format?

Partly

Reviewer Expertise:

Rehabilitation Medicine and Physiotherapy

I confirm that I have read this submission and believe that I have an appropriate level of expertise to state that I do not consider it to be of an acceptable scientific standard, for reasons outlined above.

# Reviewer report: Reviewer response for version 2

**DOI:** 10.5256/f1000research.164215.r260277

**Published:** 2024-04-24

**Authors:** Givago Silva Souza

**Affiliations:** 1 Federal University of Pará, Belém, Brazil

No more comments. All my issues were addressed.

Is the study design appropriate for the research question?

Yes

Is the rationale for, and objectives of, the study clearly described?

Yes

Are sufficient details of the methods provided to allow replication by others?

Yes

Are the datasets clearly presented in a useable and accessible format?

Not applicable

Reviewer Expertise:

Digital Health, Balance, Mobility

I confirm that I have read this submission and believe that I have an appropriate level of expertise to confirm that it is of an acceptable scientific standard.

# Reviewer report: Reviewer response for version 1

**DOI:** 10.5256/f1000research.151304.r235562

**Published:** 2024-09-16

**Authors:** Qurba Kiran

**Affiliations:** 1 Shalamar Institute of Health Sciences, Lahore, Pakistan

This study focuses on two key rehabilitation techniques: Modified Constraint-Induced Movement Therapy (mCIMT) and the Motor Relearning Program (MRP) applied to improve balance and gait in patients with sub-acute hemiplegic stroke. Hemiplegic stroke causes significant impairments in motor functions, especially affecting one side of the body, resulting in reduced mobility and balance. The study compares the effectiveness of these two interventions in enhancing motor recovery in the lower limbs. The findings of this comparative study may have direct implications on physical therapy practices. Clinics and rehabilitation centers can adopt more targeted approaches to improve outcomes for stroke survivors based on the results. The article highlights a comparative approach to two evidence-based rehabilitation techniques, contributing to the body of knowledge on effective stroke rehabilitation strategies, particularly in improving gait and balance in sub-acute hemiplegic stroke patients. The results may guide clinicians in selecting the most appropriate intervention for patient-specific needs.

*•    Baseline comparability need to be addressed*

*•    Revise the potential benefits and potential risks*

*•    Add some operational definitions of terminologies which are used in title*

*•    References should be revised as more than five years old references are not encouraged*

*•    Add some literature regarding your topic at national level*

Is the study design appropriate for the research question?

Yes

Is the rationale for, and objectives of, the study clearly described?

Partly

Are sufficient details of the methods provided to allow replication by others?

Yes

Are the datasets clearly presented in a useable and accessible format?

Not applicable

Reviewer Expertise:

Neuromuscular Physical therapy

I confirm that I have read this submission and believe that I have an appropriate level of expertise to confirm that it is of an acceptable scientific standard.

# Reviewer report: Reviewer response for version 1

**DOI:** 10.5256/f1000research.151304.r235555

**Published:** 2024-03-20

**Authors:** Givago Silva Souza

**Affiliations:** 1 Federal University of Pará, Belém, Brazil

**Abstract:**

- Please indicate the names of the groups in the abstract.

**Introduction:**

- It would be important to write a paragraph about balance and mobility in hemiplegic patients and the consequences that their losses could bring to the patients.

**Methods:**

- I suggest including the use of accelerometry to evaluate the static balance and instrumented Timed Up and Go test.

Is the study design appropriate for the research question?

Yes

Is the rationale for, and objectives of, the study clearly described?

Yes

Are sufficient details of the methods provided to allow replication by others?

Yes

Are the datasets clearly presented in a useable and accessible format?

Not applicable

Reviewer Expertise:

Digital Health, Balance, Mobility

I confirm that I have read this submission and believe that I have an appropriate level of expertise to confirm that it is of an acceptable scientific standard, however I have significant reservations, as outlined above.

# Reviewer report: Reviewer response for version 1

**DOI:** 10.5256/f1000research.151304.r235564

**Published:** 2024-02-08

**Authors:** Ingela Marklund

**Affiliations:** 1 Umeå universitet, Umeå, Sweden; 2 Centre for Clinical Research and Education, Region Värmland, Karlstad, Sweden

Thank you for the opportunity to review this study “Lower limb rehabilitation using modified constraint-induced movement therapy and motor relearning program on balance and gait in sub-acute hemiplegic stroke: a comparative study”. The authors aimed to evaluate the effect of mCIMT in addition to conventional therapy on balance and gait, the effectiveness of MRP along with conventional therapy on balance and gait and compare mCIMT and the MRP in improving balance and gait in sub-acute stroke patients. The topic is interesting and there is a lack of studies regarding mCIMT.

Introduction

 When crosschecking the references, I have doubts about whether the cited articles can really be used to make these statements (ref. 1-3, 6, 8, and 9). Please check the references and make sure that the reference really refers to a statement that can be seen as a result of the referenced work. The reference 7 and 10 are placed before the sentence instead of after and I lack a reference for conventional therapy in section three and for mCIMT and MRP in section four.

 Conventional therapy needs further explanation and a reference, what is it in your context, I doubt it is the same over the world. The sentence, “there are several anatomical and functional abnormalities that are followed by hemiplegia”, please explain what you mean with several anatomical abnormalities?.

 CIMT comprises four components: i) intensive training of the more-affected arm; ii) training with a behavioral technique called shaping; iii) the ‘transfer package’, where problem-solving is used to overcome perceived barriers to more-affected arm uses in ADL; and iv) restraint of the less-affected arm for 90% of waking hours during the treatment period (Ref 1). Shaping is also used in Lower-extremity CIMT and I miss this part in the present study. The phenomenon “learned nonuse” is more commonly used according to CIMT for the upper extremity and also used in your reference 11 and 12.

 Section five is a duplication of section four.

 The study aims are clearly described.

Methods

 The section Protocol is clearly described but the references for extended data are not in numerical order and I can not clearly understand if it is two or three assessments for outcome measures. Two in the text but three marks (x) in figure 2 (outcomes variables).

 Inclusion and Exclusion criteria are well written and does not need to be adjusted.

 Section Interventions, please describe the size of the intervention’s groups, are all 17 receiving the intervention at the same time or in smaller groups or individual? Did the principal investigator conduct both intervention and assessment? This is not clearly described and if so how can it affect the results?

 The interventions mCIMT, MRP and conventional therapy needs to be explained in more details to provide the opportunity for other researchers to replicate the study. Not only which exercise but also dose and intensity. Please describe what 10 minutes of transfer package includes. I also miss in the introduction how you came to the conclusion that 30 minutes a day for six weeks is enough? CIMT is often conducted six hours a day for two weeks and in your reference mCIMT was conducted 120 minutes a day for two weeks.

 Section Outcome measures, please use references with validity and reliability tests for the stroke population when possible. For example, BBS (Ref 2), 10MWT (Ref 3 or Ref 4), and DGI (Ref 5).

 Section Sample size calculation, I don’t understand. You wrote “we used the previously determined effect size in percentage from the RCT” but in the reference you referred to has not used BBS? Please explain where the data for the BBS you use in the calculation comes from. Is 17 in each group enough to show, as you write in the discussion part "to evaluate which is more successful while looking at the effectiveness of these therapies in sub-acute stroke patients" In the EXCITE trial (Ref 6), there were 106 and 116 in each arm. 

 Section Data collection methods and Data management are well written and I have no more questions except of the need of clarification if it is two or three assessments as I mention earlier.

 Section Statistical analysis, These variables has not been explained before, please move from statistical section to another, for example Protocol and describe the characteristics you will collect and from where, medical journals or examination? Demographic variables such as age and gender and physical examination (height, weight, BMI), laboratory parameters (haemoglobin, Erythrocyte Sedimentation Rate(ESR), Liver Function Test (LFT), Kidney Function Test (KFT)), and vitals (respiratory rate, pulse rate, systolic blood pressure, diastolic blood pressure and SpO2 levels) will be collected.

 I have no further questions or comments and look forward to following the results and discussion in the present study.

Is the study design appropriate for the research question?

Yes

Is the rationale for, and objectives of, the study clearly described?

Partly

Are sufficient details of the methods provided to allow replication by others?

No

Are the datasets clearly presented in a useable and accessible format?

Partly

Reviewer Expertise:

Rehabilitation Medicine and Physiotherapy

I confirm that I have read this submission and believe that I have an appropriate level of expertise to confirm that it is of an acceptable scientific standard, however I have significant reservations, as outlined above.

## References

[1] : The functional significance of cortical reorganization and the parallel development of CI therapy. *Front Hum Neurosci* .2014;8: 10.3389/fnhum.2014.00396 396 10.3389/fnhum.2014.00396 25018720 PMC4072972

[2] : The Balance Scale: reliability assessment with elderly residents and patients with an acute stroke. *Scand J Rehabil Med* .1995;27(1) :27-36 7792547

[3] : Mobility after stroke: reliability of measures of impairment and disability. *Int Disabil Stud* .1990;12(1) : 10.3109/03790799009166594 6-9 10.3109/03790799009166594 2211468

[4] : Test-retest reliability and minimal detectable change of gait speed in individuals undergoing rehabilitation after stroke. *J Neurol Phys Ther* .2008;32(1) : 10.1097/NPT0b013e31816593c0 8-13 10.1097/NPT0b013e31816593c0 18463550

[5] : Reliability and validity of the dynamic gait index in persons with chronic stroke. *Arch Phys Med Rehabil* .2007;88(11) : 10.1016/j.apmr.2007.08.109 1410-5 10.1016/j.apmr.2007.08.109 17964880

[6] : Effect of constraint-induced movement therapy on upper extremity function 3 to 9 months after stroke: the EXCITE randomized clinical trial. *JAMA* .2006;296(17) : 10.1001/jama.296.17.2095 2095-104 10.1001/jama.296.17.2095 17077374
